# Strain modulation of TaO_4_ planarity in tantalates ultrathin films: surface states engineering

**DOI:** 10.1038/s41598-020-64315-7

**Published:** 2020-05-08

**Authors:** Guilherme Ribeiro Portugal, Jeverson Teodoro Arantes

**Affiliations:** Center for Engineering, Modeling and Applied Social Science (CECS), Federal University of ABC (UFABC), Santo André, São Paulo Brazil

**Keywords:** Nanoscale materials, Computational nanotechnology, Surfaces, interfaces and thin films, Two-dimensional materials, Electronic structure

## Abstract

Ultrathin films of perovskites have attracted considerable attention once they fit in numerous applications. Over the years, controlling and tuning their properties have been attainable when biaxial strain is applied. Through *ab initio* DFT calculations, (110) ultrathin (Na,K)TaO_3_ films were submitted to biaxial tensile and compressive strain. Intrinsically, surface Ta shallow states emerge into the bandgap since the (110) cleavage breaks its octahedral symmetry to create TaO_4_ units. Removal of ligands along the *x*-*y* plane stabilizes *d*_*x*_^2^_*-y*_^2^ orbitals, which decrease in energy due to lower electrostatic repulsion. Such stabilization is maximized when biaxial tensile increases the TaO_4_ planarity towards a square planar symmetry. Accordingly, the corresponding electronic levels move further into the bandgap. Conversely, compressive biaxial strain intensifies electrostatic repulsion, closing the TaO_4_ tetrahedra, and surface states move to higher energy zones. The reported strain-driven modulation might be applied in different applications, as photocatalysis, ferroelectricity, and spintronics.

## Introduction

ABO_3_ perovskite structures have been broadly studied over the last decades, mostly because they constitute a family of oxides widely found in solid-state inorganic chemistry, but also because they fit into numerous technological applications^1–3^. Due to their compositional flexibility, cleaving or growing an ABO_3_ crystal in different crystallographic directions gives rise to surface planes that have atomic terminations of varying stoichiometry. Accordingly, a whole range of particular electronic structures might be found, which will govern highly sensitive local properties^4^. As a matter of fact, crystal facet engineering has been essential for the controlled improvement of physical and chemical properties, especially in nanostructures^5^. In catalytic processes, one of the main fields for perovskite applications, facet selectivity has been greatly investigated as it directly affects the efficiency of nanostructured catalysts^6,7^.

In an ideal perovskite, a cubic lattice is composed of a corner-sharing BO_6_ octahedra framework whose dodecahedral interspaces are filled with A-site cations. Breaking such crystal will eventually expose lower coordination BO_x=3,4,5_ units on the surface, which is the major responsible for the electronic structure of cleaved crystals. Much is known about how BO_6_ octahedra rotation and tilting affect the material’s properties^8–10^, sometimes inducing particular features as ferroelectricity^11^. However, accurate information regarding other BO_x_ units is necessary to a complete understanding of physical and electronic properties of perovskites’ surfaces.

Of particular interest, meaningful properties have been attributed to BO_4_ groups. Molybdates having MoO_4_ units presented enhanced O_2_ evolution activity, which means their valence bands (VB) are well-aligned with water oxidation potentials^12^. Recently, (100) NaTaO_3_ orthorhombic slabs have also shown similar alignment thanks to localized TaO_4_ energy states^13^. Interestingly, there is strong evidence that the excited energy is localized in isolated BO_4_ tetrahedra inside scheelite structures^12^. In sillenites, specific transmittance and absorption peaks in the visible region have been observed and associated with discrete BO_4_ groups^14^. Furthermore, in mullite-type Bi_2_B_4_O_9_ (B = Al^3+^, Ga^3+^) materials, unbalanced Mulliken charges and symmetry breaking related to BO_4_ tetrahedra assisted the charge separation positively, increasing their photocatalytic reactivity^15^. Although there are a few records of straight relationships between the BO_4_ units and electronic structure, further investigation on how its possible configurational geometry might influence the material’s electronic properties is required so that their modulation can be feasible.

Among recurrently synthesized nanostructures, thin and ultrathin films allow fine surface science investigations since they have approaching bulk-like characteristics^16^. Such nanostructured materials may have their properties tuned and controlled when strain (stress) is induced^17,18^. The effects of biaxial strain have been frequently explored due to the surprising responses obtained when it comes to properties’ modulation, which ranges from induction of dielectric anomalies^19^ to bandgap engineering^20^. To control the amount of induced strain, substrates with different mismatches with the films are usually employed^21^. The application of an electric field to a piezoelectric substrate has also been used to induce biaxial strain and modulate specific properties^22^. Additionally, developed synthesis methodologies have granted refined control of surface particle size as well as surface area and morphology in thin films, not to mention the fact that characterization techniques that are usually difficult to be implemented for nanoparticles (scanning probe microscopy, for instance) can be readily used in 2-D materials^23,24^. Therefore, studying the influence of surface BO_x_ arrangements on ultrathin films should provide clear evidence of their role in controlling electronic properties.

Recently, several experimental reports have driven their attention to the synthesis of ABO_3_ thin films, such as NaTaO_3_^24,25^ and KTaO_3_^26–28^, on different substrates and heterostructures. Amazingly, angstroms-thick perovskites ultrathin films have already been achieved^29–32^, which supports reliable theoretical and experimental comparisons. Furthermore, the modulation of perovskite properties by means of biaxial strain is also widely reported and highly relevant to various applications. Magnetic, electrical, and transport properties, for example, react significantly to strain and can therefore be tuned^33,34^. Strain-induced lattice deformation (octahedral tilting)^35^ as well as photoluminescence features^36^ have also shown considerable changes upon biaxial strain. Here, through *ab initio* density functional theory^37,38^, we have systematically studied the effects of biaxial strain on (110) cubic (Na,K)TaO_3_ ultrathin films, emphasizing how the geometric arrangement of surface-exposed TaO_4_ tetrahedra influence their electronic structure. Shallow surface states of Ta e_g_ orbitals are located below the conduction band (CB) and can be pushed either into or out of the bandgap as strain is applied and TaO_4_ planarity varies. Biaxial tensile strain increases Ta-O bond distances and the unit planarity, stabilizing e_g_ orbitals and shifting surface states into the gap. The opposite movement is produced when biaxial compressive strain reduces Ta-O bond distances and makes TaO_4_ units less planar. To the best of our knowledge, the aforesaid structural-electronic connection has not been reported hitherto and allows bandgap states engineering, which is desirable in many different fields.

## Computational Details

Our calculations have run on Vienna Ab Initio Simulation Package (VASP)^39,40^, with electron-ion interactions described by projector augmented-wave (PAW)^41^ pseudopotentials. For electrons exchange-correlation interactions, the generalized gradient approximation (GGA) within the PBE functional^42^ has been used. To sample the Brillouin zone, **k**-points meshes were generated following the Monkhorst-Pack^43^ scheme. During geometry optimizations, plane-wave cutoff energy of 520 eV and electronic energy convergence for self-consistent iterations of 10^−8^ eV were set, aiming at accurate results.

**Figure 1:**
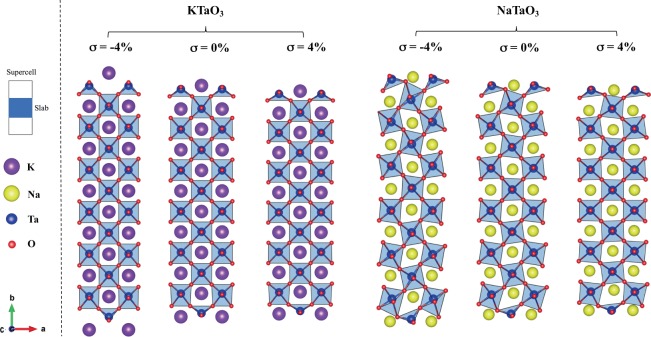
Optimized structures of ultrathin KTO (left panel) and NTO (right panel) films. For each film, the structure having the bulk lattice constant (*σ* = 0%) is in the center, whereas the maximum compression (*σ* = −4%) is on the left, and the maximum tensile (*σ* = 4%) is on the right. In both cases, surface TaO_4_ tetrahedra become more planar under biaxial tensile and less planar under biaxial compressive strain.

In order to have a reference from which ultrathin films could be built using a slab approach, we have first optimized KTaO_3_ (KTO) and NaTaO_3_ (NTO) cubic *Pm*$\bar{3}$*m* bulk structures, performing 11 × 11 × 11 **k**-points integrations. The equilibrium lattice parameters were *a*_0_^KTO^ = 4.04 Å and *a*_0_^NTO^ = 3.98 Å, which are in agreement with experimentally reported values^44,45^. Typical semiconductor band structures (Fig. 1S) with a Γ-R indirect bandgap of 2.07 eV (KTO) and 2.26 eV (NTO) have been obtained. Despite being well-known that GGA underestimates the bandgap when compared to experimental data^45,46^, it agrees with other similar theoretical studies^47,48^.

Ultrathin KTO and NTO films were constructed by replicating the bulk and cleaving it in the (110) direction. Thus, 12 layers thick (~31 Å) films that expose such a surface were relaxed with a 15 Å vacuum to minimize image self-interactions from periodic boundary conditions. The **k**-points integration was performed up to a 5 × 1 × 5 grid. To simulate in-plane epitaxial strain, the interplane *x*-*z* lattice constants were changed up to −4% and 4% for compressive and tensile strain, respectively. We designated the strain as *σ* = $[({\alpha }_{KTO,NTO}-{a}_{0})/{a}_{0}]\times 100$, where *α*_KTO,NTO_ is the strained lattice parameter and *a*_0_ is the bulk optimized lattice parameter.

## Results and Discussion

**Figure 2:**
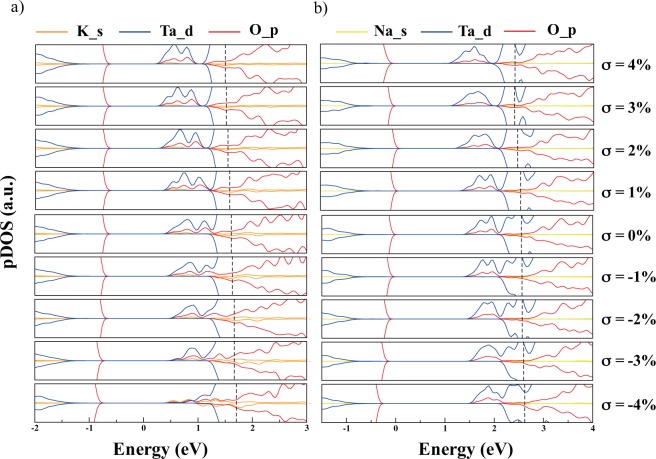
Projected density of states of KTO (**a**) and NTO (**b**) ultrathin films. Surface states are clearly seen near the conduction band bottom of the films. When compression is induced, *σ* < 0%, such levels are pushed towards the conduction band. Oppositely, when tensile is applied, *σ* > 0%, they move into the bandgap. Black dashed lines represent the Fermi levels, and the plots were aligned according to their electrostatic potential.

According to the equilibrium structure of the films, biaxial strain may induce changes in both structural (bond angles, bond distances, crystallinity, etc.) and electronic properties (bandgap, electronic states, Fermi level, etc.). Strain-free, tensile *σ* = 4% (*α*_KTO_ = 4.02 Å, *α*_NTO_ = 4.14 Å), and compression *σ* = −4% (*α*_KTO_ = 3.88 Å, *α*_NTO_ = 3.82 Å) converged structures are displayed in Fig. 1. For both KTO and NTO, strain-free films are more stable than those under tensile and compressive strain (Fig. 2S). Despite exposing polar terminations with non-zero formal charges, no surface reconstruction has been observed. Therefore, an electronic reconstruction mechanism will compensate the polarity induced by the cleavage. KTO structures retain their cubic lattice and relaxation occurs by changes in the interlayer spacing along the *y*-axis. NTO films, in turn, relax by similar mechanisms but also allow the rotation of internal TaO_6_ and surface TaO_4_ units. Note that under biaxial compression, the film acquires a more orthorhombic character while the bulk cubic group spacing can be partially recovered under tensile. A closer look at surface layers reveals a common feature presented by both films upon relaxation. Under biaxial compression (*σ* < 0%), Ta-O bond distances decrease and TaO_4_ tetrahedral units become less planar, whereas under biaxial tensile strain (*σ* > 0%), Ta-O bond distances increase and so does their planarity. We here call planarity (*P*_*L*_) an average of in-plane O-Ta-O angles so that the upper planarity limit corresponds to both *x* and *z* plane angles being 180°.

**Figure 3:**
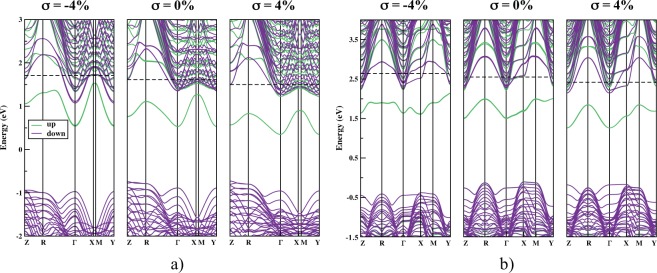
Band structure of KTO (**a**) and NTO (**b**) ultrathin films. In each panel, the strain-free case is in the middle, maximum compression is on the left, and maximum tensile in on the right. Surface up states near the CB are confirmed and their change when *σ* ≠ 0% is evident. Black dashed lines represent the Fermi levels, and the plots were aligned according to their electrostatic potential.

Since surface states are crucial in the electronic structure of low dimension materials, we have plotted the projected density of states on the potassium (K) *s*, oxygen (O) *p*, and tantalum (Ta) *d* orbitals in Fig. 2. As in bulk, the valence band maximum consists of O 2*p* states and the conduction band minimum is mostly Ta 5*d* levels with a slight O 2*p* contribution, which indicates *d*-*p* (*π*) hybridization states. Nevertheless, a metallic character is observed in all films as a consequence of their polarity, which induces electronic reconstruction by filling up conduction states. It is interesting to note that there are spin up well-localized energy states near the CB which appear to be shallow and within the bandgap, especially under tensile. The layer resolved projected density of states (LRPDOS, Fig. 3S) confirms that such states are derived from surface TaO_4_ units. Under compressive conditions where *σ* < 0%, these states migrate to higher energy regions, eventually entering the CB energy zones. When tensile acts on the system, *σ* > 0%, the opposite behavior is observed and they move to lower energy zones within the material’s bandgap.

**Figure 4:**
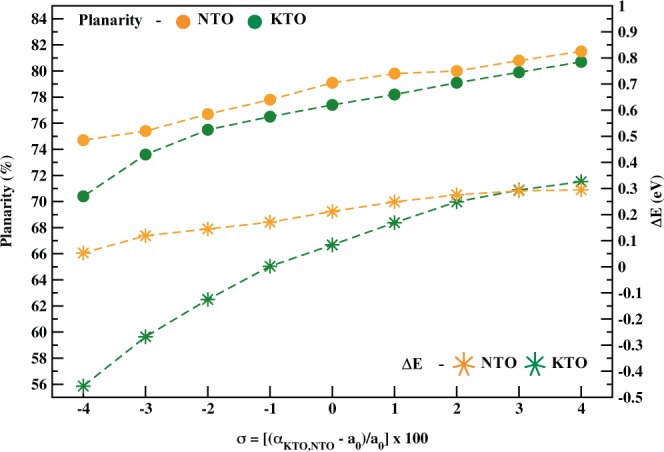
Surface TaO_4_ planarity (*P*_*L*_) and the energy difference between shallow Ta surface states and the CB (ΔE) as functions of biaxial strain (*σ*). On the left *y*-axis, *P*_*L*_ (bullets) behaves almost linearly with strain and, intrinsically, NTO has surface TaO_4_ units more planar than KTO ones. On the right *y*-axis, ΔE (stars) increases up to a limit around 0.30 eV. Negative values of ΔE indicate surface states/CB overlap.

The band structure of the films (Fig. 3) helps in describing such TaO_4_ surface states, pointing out their energy difference (ΔE) regarding the CB. A complete plot of both *P*_*L*_ and ΔE as functions of *σ* can be seen in Fig. 4 (all the related data is in Table 1S). It turns out that for *σ* = 0% both KTO and NTO surface states are already separated from the CB by a total of ΔE = 0.08 eV for the former and ΔE = 0.21 eV for the latter, which characterizes them as shallow states within the bandgap. The planarity values of TaO_4_ surface tetrahedra are *P*_*L*_ = 77.4% (KTO) and *P*_*L*_ = 79.1% (NTO). When the tensile limit of *σ* = 4% is achieved, such planarity rises to 80.7% and 81.5%, respectively. Accordingly, ΔE increases to 0.33 eV (KTO) and 0.30 eV (NTO), which sets the levels even further into the gap. On the other hand, when the compression limit of *σ* = −4% is reached, *P*_*L*_ of surface TaO_4_ units reduces to 70.4% for KTO and 71.5% for NTO. This approximates surface states and the CB, resulting in a significant bands overlap for KTO, and reducing ΔE to 0.05 eV for NTO. The above results shall be explained in two different but complementary discussions: (i) the reason why surface Ta *d* states lie below the CB; (ii) the way biaxial strain changes the position of such states.

**Figure 5:**
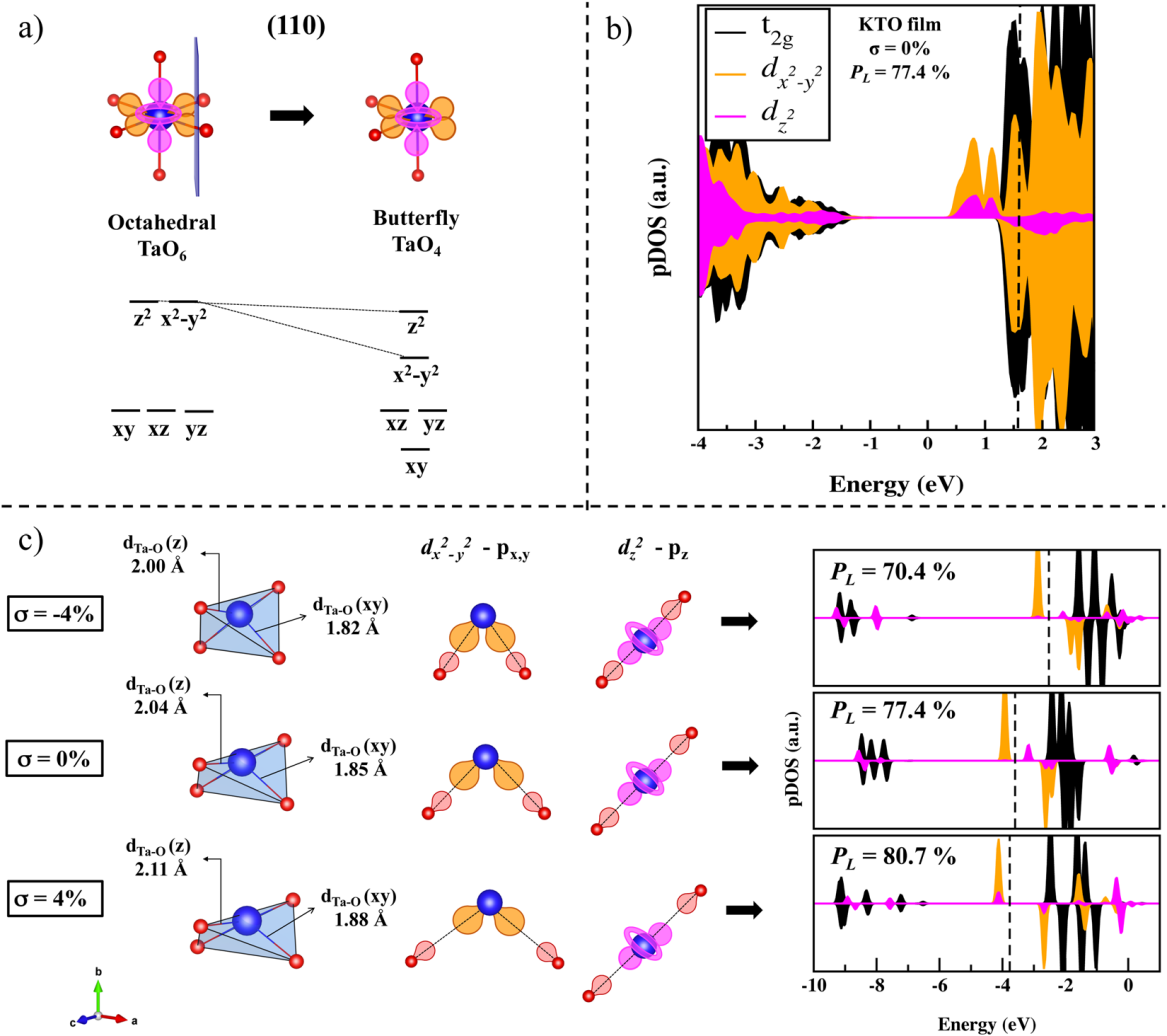
Investigation of individual TaO_4_ units and their electronic characterization: (**a**) schematic representation of how the (110) cleavage removes *cis* oxygen ligands and stabilizes e_g_ orbitals; (**b**) relaxed strain-free (*σ* = 0%) KTO film projected density of states on e_g_ and t_2g_ orbitals; (**c**) from the left to the right, structural changes such as the variation of both Ta-O bond distances and the unit planarity are induced by biaxial strain, which alters *d-p* repulsive interactions, and shift e_g_ states to lower (tensile, further stabilization) or higher (compression, destabilization) energy zones, as shown by the individual isolated TaO_4_ pDOS. Red, orange, and pink schematic orbitals refer to O *p*, Ta ${d}_{{x}^{2}-{y}^{2}}$, and Ta ${d}_{{z}^{2}}$ orbitals, respectively. Black dashed lines within pDOS plots represent the Fermi level.

As for the first, we have to consider the consequences that cleaving atomic bonds triggers on an initially TaO_6_ octahedral (O_h_) symmetry existing in bulk KTO and NTO. As part of a group five transition metal, the five-fold degenerated Ta *d* orbitals are split by the ligand field of six oxygen atoms approaching along the axes, forming a set of doubly-degenerated e_g_ (${d}_{{{\rm{z}}}^{2}}$ and ${d}_{{{\rm{x}}}^{2}-{{\rm{y}}}^{2}}$) and triple-degenerated t_2g_ (*d*_xy_, *d*_yz_, *d*_zx_) orbitals. Being oxygen a *π*-donor ligand, charge transfer occurs through *π* bonds due to lateral overlap of ligand O 2*p* and Ta t_2g_ orbitals, which causes their energy to decrease. On the other hand, e_g_ orbitals rise in energy since their interactions with ligand orbitals are mostly repulsive (σ*)^49^. Consequently, in the TaO_6_ bulk arrangement, the CB is composed by Ta t_2g_-O*p* (π*) bands followed by a higher lying set of Ta e_g_-O *p* (σ*) bands. Cleaving the crystal in order to reduce the unit coordination to TaO_4_ removes two oxygen ligands. In such cases, the absence of ligands along the axes stabilizes the corresponding e_g_ orbital, reducing their repulsive interactions^50^. Therefore, e_g_ orbitals shall lower in energy but now with the degeneracy broken since ${d}_{{z}^{2}}$ and ${d}_{{{\rm{x}}}^{2}-{{\rm{y}}}^{2}}$ will not be equally stabilized. For instance, changes in the ligand field as it moves from octahedral to a square planar symmetry are generally described as a consequence of removing two ligands along the *z*-axis, which stabilizes ${d}_{{z}^{2}}$ orbitals electrostatically and increases the energy of ${d}_{{{\rm{x}}}^{2}-{{\rm{y}}}^{2}}$ orbitals that now suffer the greatest repulsion^51^ (Fig. 4S). For the proposed thin films, the (110) cleavage removes two *cis* oxygen atoms, leaving initially ‘butterfly’ TaO_4_ complexes on the surface. Such a cleavage cut out half of ${d}_{{{\rm{x}}}^{2}-{{\rm{y}}}^{2}}$ interactions, while the main electrostatic repulsion in *z* remains the same, as illustrated in Fig. 5(a). The O-Ta-O angles may acquire variable values, changing the TaO_4_ planarity so that tetrahedral (T_d_), square planar (D_4h_), and intermediate symmetries are allowed. In the studied structures, relaxation induces intermediate values of planarity to reach the structure equilibrium, balancing electrostatic and steric interactions. Figure 5(b) reveals the e_g_ nature of surface states near the CB, confirming the stabilization of ${d}_{{{\rm{x}}}^{2}-{{\rm{y}}}^{2}}$ orbitals caused by the (110) cleavage. The small contribution of ${d}_{{z}^{2}}$ states result from a slight stabilization of its central lobe that reduces in energy when two weak antibonding interactions are removed.

As for the way that biaxial strain changes the position of surface Ta *d* states, an individual investigation of isolated TaO_4_ units is required. We have calculated TaO_4_ tetrahedral groups with different spatial arrangements in 20 Å vacuum supercells (oxygen atoms were saturated with hydrogen to mimic their coordination in both bulk and films). In addition to the configurational case of the strain-free KTO film, we have also calculated TaO_4_ units with the coordinates and hence planarity in which they are found on KTO film surfaces when *σ* = ±4%. In the left panel of Fig. 5(c), starting from the strain-free tetrahedra (*σ* = 0%, ${d}_{TaO}(xy)$ = 1.85 Å, and ${d}_{TaO}(z)$ = 2.04 Å), it is shown that biaxial tensile (*σ* = 4%) stretches d_Ta-O_ bonds to ${d}_{TaO}(xy)$ = 1.88 Å, and ${d}_{TaO}(z)$ = 2.11 Å, rising the unit planarity, whereas biaxial compression (*σ* = −4%) diminishes the atomic bond distances to ${d}_{TaO}(xy)$ = 1.82 Å, and ${d}_{TaO}(z)$ = 2.00 Å, decreasing the planarity. All bond distances of the discussed cases can be found in Table 2S. The central panel of Fig. 5(c) illustrates the weakening or strengthening of repulsive interactions between antibonding Ta e_g_-O *p* orbitals when tensile or compression is applied, respectively. The consequence in the material’s electronic structure is depicted on the right panel. The projected density of states reveals that as a (110) cleavage is performed in the cubic bulk, ${d}_{{{\rm{x}}}^{2}-{{\rm{y}}}^{2}}$ and ${d}_{{z}^{2}}$ states are stabilized and appear occupied (spin-up) below the Fermi level after structural relaxation. If biaxial tensile strain is applied in the *x-z* plane, Ta-O bonds will stretch, repulsive *d-p* interactions along such axes will decrease, and e_g_ orbitals are stabilized. Conversely, biaxial compression along *x-z* brings atoms close together, repulsive *d-p* interactions increase, and e_g_ orbitals rise in energy.

Finally, we note the fact that the electronic structure of ultrathin KTO films appears to be more sensitive to changes in surface TaO_4_ planarity than NTO films. As shown in Fig. 4 (left *y*-axis), the planarity of superficial TaO_4_ groups in both films respond very similarly to biaxial strain. Showing an essentially linear variation, the *P*_*L*_ increases under tensile and decreases under compression, following basically the same slope. On the other hand, the linear coefficient is smaller for the KTO film than for the NTO one, what indicates that the initial planarity of the former is intrinsically lower than that of the latter. Since potassium has an extra electron shell compared to sodium, and hence a larger atomic radius, the repulsion felt by the superficial K-Ta-O arrangement is greater than in the case of Na-Ta-O, which initially prevents KTO-TaO_4_ groups from being as planar as NTO-TaO_4_ ones. As the electrostatic interactions are stronger for KTO, it was expected that its electronic structure would change more sensibly upon strain, as shown in Fig. 2. The exact influence of A-site cation on this type of process is still under investigation, nevertheless.

We also suggest that there are upper and lower limits of planarity that biaxial strain is able to induce. Despite the almost linear behavior of *P*_*L*_ as a function of strain, too high tensile values stretch Ta-O to such an extent that the TaO_4_ coordination might be broken. In turn, too high compressive values maximize electrostatic and steric repulsions. For the KTO film, for instance, the repulsion at *σ* = −4% is such that potassium atoms start a exsolution-like process and are “expelled” from the surface of the film, TaO_4_ planarity values decrease drastically, and the electronic structure changes considerably. When the maximum tensile is achieved, in turn, both films tend to the similar planarity values and to the same ΔE maximum of ~0.30 eV from which greater *P*_*L*_ is only achieved if more severe strain that irreversibly destabilizes the system is applied.

## Conclusions

In conclusion, the (110) cleavage in cubic KTaO_3_ and NaTaO_3_ crystals made to obtain ultrathin films modifies the Ta *d* orbitals degeneracy and local electrostatic interactions to such an extent that shallow surface states can be manipulated by biaxial strain. Cleaving the crystal in such direction naturally induces ${d}_{{{\rm{x}}}^{2}-{{\rm{y}}}^{2}}$ orbitals stabilization and introduces them near the CB. We systematically investigated and showed that biaxial tensile or compression modifies surface TaO_4_ planarity, and therefore the energy of electronic defect states. Biaxial tensile pushes them further into the material’s bandgap while compression moves them towards the CB. As the behavior is generically the same for both different tantalates, we report a practical way of tuning their electronic properties through biaxial strain. Our results elucidate meaningful structural-electronic relationships that can be readily harnessed to control and engineer bandgap energy states. Although further investigation is required to connect such results with tactile applications, the proposed engineering may be widely desirable. For photocatalysis (the main application of NaTaO_3_ nanostructures), for instance, the alignment of CB and VB states with the water redox potentials is a major challenge and usually achieved by doping. Such strain-driven modulation may be effective and rule out the use of foreign materials. Concerning less explored applications, as spintronics, the magnetic characteristics of the system can be useful for building spin filters or similar devices.

## Supplementary information

Supplementary Information.
