# Supplementary material for: Strain modulation of TaO4 planarity in tantalates ultrathin films: surface states engineering
Source: Sci Rep. 2020 May 8;10:7828. doi: 10.1038/s41598-020-64315-7 (PMC7210982; doi:10.1038/s41598-020-64315-7)
Supplement: Supplementary file 1 — Supplementary Information. [file 41598_2020_64315_MOESM1_ESM.pdf]

Supplementary Information for

Strain modulation of TaO_4 planarity in tantalates ultrathin films: surface states engineering

Guilherme Ribeiro Portugal and Jeverson Teodoro Arantes*

Center for Engineering, Modeling and Applied Social Science (CECS), Federal University of ABC (UFABC), Santo André, São Paulo, Brazil.

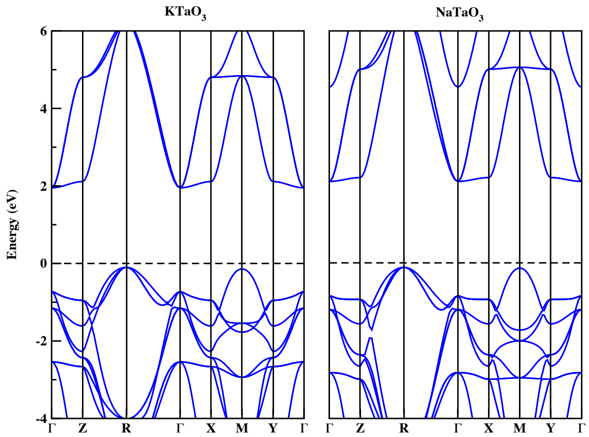

Figure 1S. Band structure of cubic $KTaO_3$ and $NaTaO_3$ bulk. Both materials present typical semiconductor band structures with Γ- R bandgaps of 2.07 and 2.26 eV, respectively. The Fermi level has been set to zero and is represented by black dashed lines.

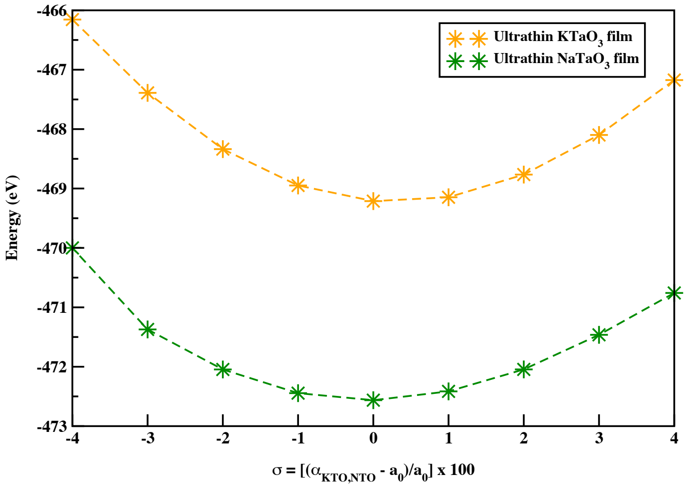

Figure 2S. Total energy dependence on biaxial strain. The energy minimum corresponds to bulk lattice parameters (σ = 0%).

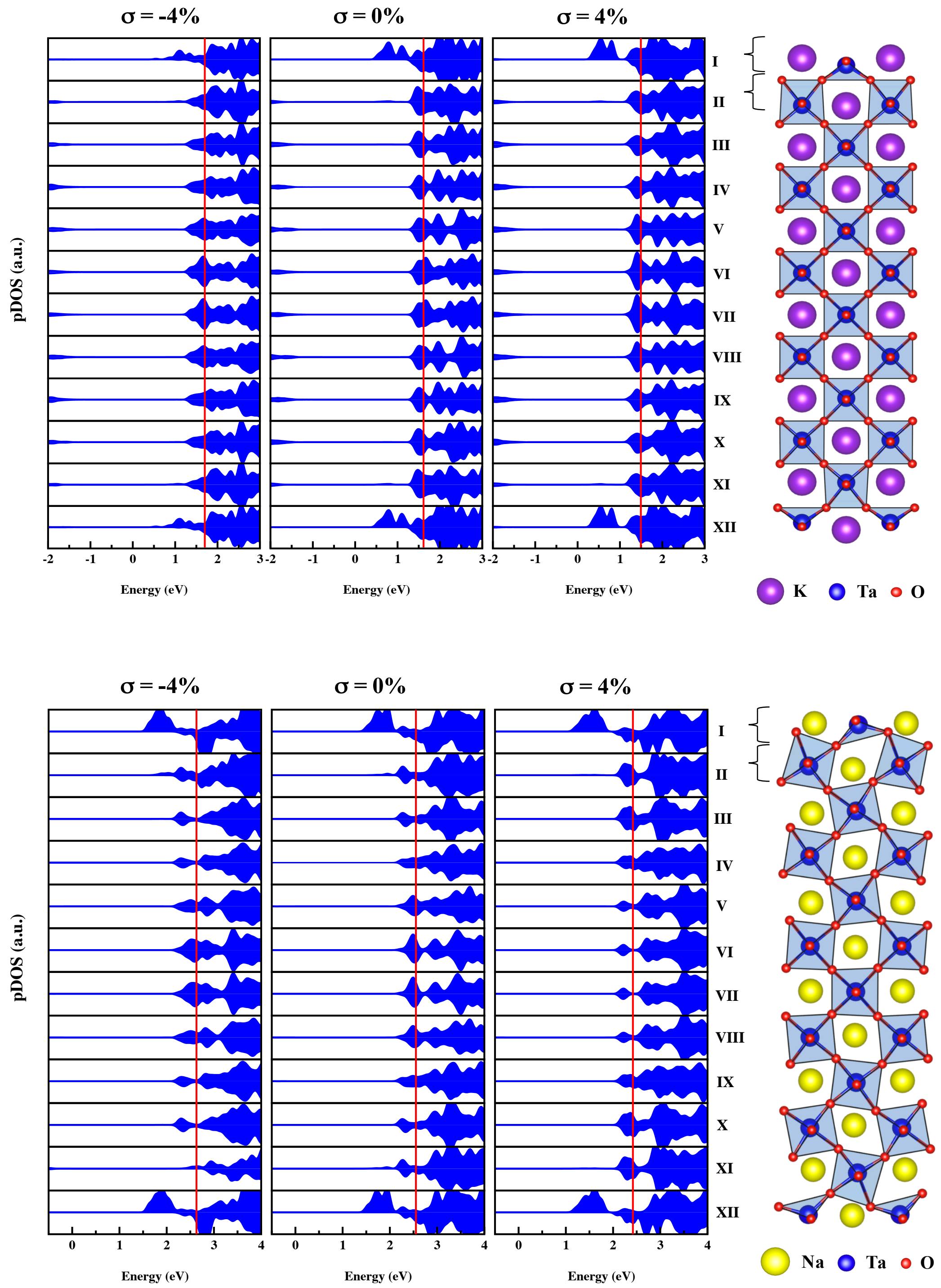

Figure 3S. Tantalum *d* orbitals layer resolved projected density of states (LRPDOS) for both ultrathin films. It reveals the surface TaO_4 character of energy levels lying right below the films' CB and confirms that they remain with the same character when compression and tensile strain limits are reached. The Fermi level is indicated by red lines, and the plots were aligned according to their electrostatic potential.

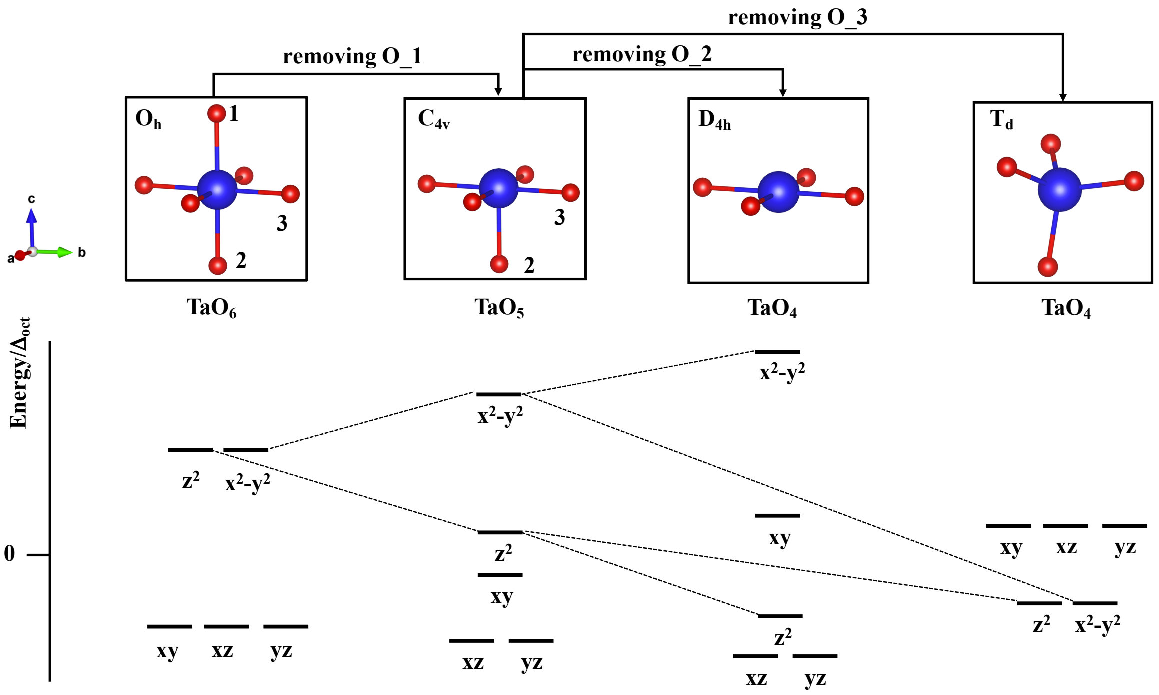

Figure 4S. Schematic representation of possible $TaO_{6,5,4}$ arrangements and their respective *d* orbital diagram. From the left to the right, starting from a complete TaO_6 octahedral O_h symmetry, e_g orbitals find themselves degenerated and with higher energy than t_{2g} ones due to electrostatic repulsion in the in-axes orbital interactions. Removing the topmost oxygen ligand (along the *z* axis), the degeneracy is broken and e_g is split in such a way that the d_{z^2} orbital is stabilized, lowering in energy for tetragonal pyramidal C_{4v} complexes. A new cleavage, which removes the bottommost oxygen (also along the *z* axis), leads to two possible configurations: i) a square planar D_{4h} symmetry that promotes further stabilization of the d_{z^2} orbital; ii) a tetrahedral T_d symmetry that equally stabilizes d_{z^2} and $d_{x^2-y^2}$ orbitals, resuming the initial e_g degeneracy.

Table 1S. Lattice constants (α), planarity (P_L), and energy difference between shallow surface states and the CB (ΔE) upon biaxial strain for $KTaO_3$ (KTO) and $NaTaO_3$ (NTO).

	KTO			NTO		
Strain	α (Å)	P_L (%)	ΔE (eV)	α (Å)	P_L (%)	ΔE
4%	4.20	80.7	0.326	4.14	81.5	0.295
3%	4.16	79.9	0.294	4.10	80.8	0.291
2%	4.12	79.1	0.249	4.06	80.0	0.276
1%	4.08	78.2	0.169	4.02	79.8	0.249
0%	4.04	77.4	0.084	3.98	79.1	0.212
-1%	4.00	76.5	0.02	3.94	77.8	0.171
-2%	3.96	75.5	-0.125	3.90	76.7	0.145
-3%	3.92	73.6	-0.268	3.86	75.4	0.119
-4%	3.88	70.4	-0.457	3.82	74.7	0.053

Table 2S. Metal-oxygen bond distances ($d_{Ta\text{-}O}$, Å) of surface TaO_4 units for both $KTaO_3$ (KTO) and $NaTaO_3$ (NTO) ultrathin films. There are two Ta-O bonds along de *x-y* plane, denoted $d_{Ta\text{-}O}$ *(xy, n° 1,2)*, and two along de *z* axis, denoted $d_{Ta\text{-}O}$ *(z, n° 1,2)*. For comparison, strain-free (σ = 0%) as well as the strain limits (σ = ± 4%) are displayed.

		KTO			NTO	
Bond distance (Å)	σ= -4%	σ= 0%	σ= 4%	σ= -4%	σ= 0%	σ= 4%
$d_{Ta-O}(xy, n^o1)$	1.828	1.856	1.871	1.976	2.009	1.907
$d_{Ta-O}(xy, n^o2)$	1.829	1.857	1.874	1.972	2.021	2.261
$d_{Ta-O}(z, n^o1)$	2.001	2.041	2.112	1.850	1.891	1.873
$d_{Ta-O}(z, n^o2)$	2.002	2.042	2.112	1.887	1.873	1.891
